# A Molecularly Imprinted Electrochemical Sensor for Carbendazim Detection Based on Synergy Amplified Effect of Bioelectrocatalysis and Nanocomposites

**DOI:** 10.3390/polym17010092

**Published:** 2025-01-01

**Authors:** Wenjing Lian, Xinyu Zhang, Yongbin Han, Xintong Li, Hongyun Liu

**Affiliations:** 1 Department of Applied Chemistry, College of Basic Science, Tianjin Agricultural University, Tianjin 300392, China; hhw18322436194@163.com (X.Z.); 18526048625@163.com (Y.H.); 13602107686@163.com (X.L.); 2 Key Laboratory of Radiopharmaceuticals, Ministry of Education, College of Chemistry, Beijing Normal University, Beijing 100875, China

**Keywords:** electrochemical sensor, molecularly imprinted polymer, bioelectrocatalysis, graphene oxide, gold nanoparticles, carbendazim

## Abstract

The highly selective and sensitive determination of pesticide residues in food is critical for human health protection. Herein, the specific selectivity of molecularly imprinted polymers (MIPs) was proposed to construct an electrochemical sensor for the detection of carbendazim (CBD), one of the famous broad-spectrum fungicides, by combining with the synergistic effect of bioelectrocatalysis and nanocomposites. Gold nanoparticle-reduced graphene oxide (AuNP-rGO) composites were electrodeposited on a polished glassy carbon electrode (GCE). Then the MIP films were electropolymerized on the surface of the nanolayer using CBD as the template molecule and o-phenylenediamine (OPD) as the monomer. The detection sensitivity of CBD on the heterogeneous structure films was greatly amplified by AuNP-rGO composites and the bioelectrochemical oxidation of glucose, which was catalyzed by glucose oxidase (GOD) with the help of mediator in the underlying solution. The developed sensor showed high selectivity, good reproducibility, and excellent stability towards CBD with the linear range from 2.0 × 10^−9^ to 7.0 × 10^−5^ M, and the limit of detection (LOD) of 0.68 nM (S/N = 3). The expected system would provide a new idea for the development of simple and sensitive molecularly imprinted electrochemical sensors (MIESs).

## 1. Introduction

Molecular imprinting technology is a molecular recognition technology for synthesizing molecularly imprinted polymers (MIPs) that have specific recognition effects on target analytes [1,2,3,4]. In recent years, MIPs have been widely applied in various fields such as sensors, chromatographic analysis, enzyme mimics, food detection, and environmental monitoring due to their excellent properties including low cost, storage stability, easy preparation, high selectivity, and good mechanical strength [5,6,7]. Molecularly imprinted electrochemical sensors (MIESs), which combine the excellent specific selectivity of MIPs with a simple and rapid electrochemical detection method, have been extensively used in analytical chemistry [8,9,10]. However, the limited number of specific recognition sites after template molecule removal and the relatively weak conductivity of MIPs have significantly restricted the detection sensitivity of MIESs [11,12]. Therefore, it is of great application value to utilize simple and efficient methods to enhance the detection sensitivity of MIESs and achieve rapid and sensitive determination of target analytes.

In order to improve the detection sensitivity of MIESs, a commonly used pathway is to utilize the enhancing effect of nanomaterials with excellent properties such as large specific surface areas and good electrical conductivity [13,14,15,16,17,18]. For example, Feng et al. constructed an MIES based on nitrogen and sulfur-doped hollow Mo_2_C/C spheres (N, S–Mo_2_C) for CBD detection [18]. The MIPs were prepared in situ on the surface of N, S–Mo_2_C/GCE by an electropolymerization method with o-phenylenediamine (OPD) as the functional monomer, and the sensor exhibited high sensitivity and favorable selectivity for the detection of CBD. Graphene and gold nanoparticles (AuNPs) have attracted great attention in the field of electrochemical sensing due to their excellent electrical conductivity, a large specific surface area, excellent biocompatibility, and remarkable mechanical properties [19,20,21,22,23]. In addition, by using the electrochemical deposition method, under an appropriate potential, the reduction of chloroauric acid and graphene oxide (GO) could be achieved in one step under normal temperature and pressure conditions. Therefore, the AuNP-rGO composites could be directly deposited on the surface of the electrode, with the advantages of high efficiency and controllability in the preparation process [24,25,26]. Due to the synergistic signal amplification effect of rGO and AuNP, AuNP-rGO composites exhibit better electrochemical properties than a separate nanomaterial.

Another effective pathway for enhancing the detection sensitivity of MIESs is to utilize the enzymatic reaction. For example, Li’s group has conducted extensive research on enhancing the detection sensitivity of MIES by utilizing the catalytic amplification effect of enzymes [27,28]. Their amplification approach came from the competition for the recognition sites within the MIP films between the template molecule and the enzyme-labeled template. Lian et al. have reported an MIES that ingeniously combined the specific selectivity of MIPs with the amplification effect of enzymatic bioelectrocatalysis in solution [12]. In their work, a simple bioelectrocatalytic system of horseradish (HRP)-H_2_O_2_-K_3_[Fe(CN)_6_] was introduced to efficiently improve the detection sensitivity of kanamycin with MIP films. However, as far as we know, the construction of MIESs by utilizing the synergistic amplification effect of nanocomposites and bioelectrocatalysis has not been reported.

**Scheme 1:**
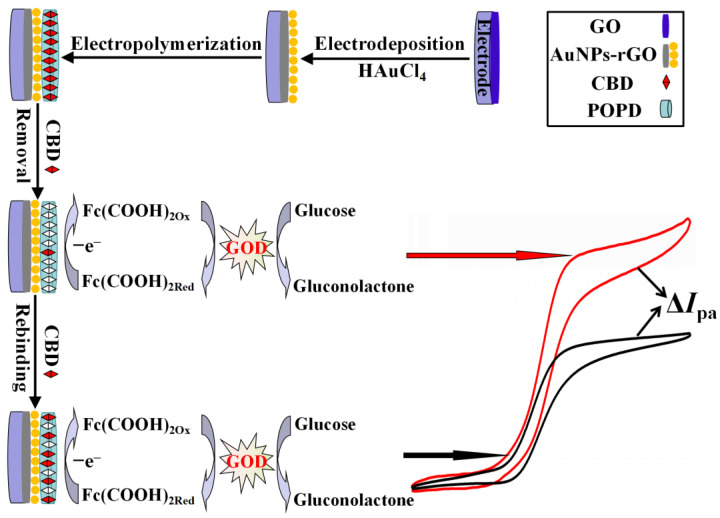
Schematic illustration of the fabrication process of the CBD-imprinted sensors.

In the present work, a sensitive MIES was established for carbendazim (CBD) detection based on the synergy amplified effect of bioelectrocatalysis and nanocomposites. As a broad-spectrum fungicide pesticide, small molecule CBD is commonly used as a fungicide for cereal crops, fruits, vegetables, and other plants [29,30]. The construction process of the CBD-imprinted sensor is illustrated in Scheme 1. The AuNP-rGO composite layer was firstly prepared on the surface of the GCE electrode by one-step electrodeposited method. The AuNP-rGO nanocomposites could effectively enhance the electrochemical response of the sensor. Functional monomer o-phenylenediamine (OPD) was electropolymerized on the surface of the AuNP-rGO/GCE electrode with CBD as the template to prepare CBD-recognized MIP films. Electroactive ferrocene dicarboxylic acid (Fc(COOH)_2_) was used as the probe in the cyclic voltametric (CV) measurements. The difference of CV oxidation peaks (Δ*I_pa_*) of Fc(COOH)_2_ in the solution between MIP film electrodes after CBD removal and CBD-rebinding could be used to determine CBD quantitatively. In order to further enhance the detection sensitivity of the sensor, the amplification effect of the glucose oxidase (GOD)-glucose system was introduced due to the bioelectrocatalytically oxidized glucose by GOD and mediated by Fc(COOH)_2_. This imprinting-typed electrochemical sensor showed a low limit of detection (LOD), high selectivity, and excellent stability towards CBD and it was successfully used to detect CBD in real samples. The present work was expected to offer a novel concept for the advancement of convenient and sensitive MIESs.

## 2. Materials and Methods

### 2.1. Chemicals

Carbendazim (CBD), glucose oxidase (GOD, E.C. 1.1.3.4, type VII, MW ≈ 160,000, 250 units g^−1^), chloroauric acid (HAuCl_4_), and 1,1′-ferrocenedicarboxylic acid (Fc(COOH)_2_) were purchased from Sigma-Aldrich (St. Louis, MO, USA). Methanol, o-phenylenediamine (OPD), acetic acid, and sodium acetate were obtained from TCI Chemicals (Tokyo, Japan). Graphene oxide (GO), potassium ferricyanide (K_3_[Fe(CN)_6_]), glucose, potassium ferrocyanide (K_4_[Fe(CN)_6_]), thiabendazole (TBZ), thiophanate methyl (TPM), and 2-aminobenzimidazole (2-ABZ) were purchased from Shanghai Meyer Biochemical Technology (Shanghai, China). All chemicals were analytical grade and all aqueous solutions were prepared with an ultra-pure water purification system (18.2 MΩ cm).

### 2.2. Instruments

In this work, electrochemical measurements of CV and electrochemical impedance spectroscopy (EIS) were performed using a CHI 660E electrochemical workstation (Chenhua, Shanghai, China). A three-electrode system was used, which was composed of a platinum wire as auxiliary electrode, a saturated calomel electrode (SCE) as reference electrode and a modified GCE electrode as working electrode. The EIS measurements were performed in 5 mM Fe(CN)_6_^4−/3−^ (1:1, containing 0.1 M NaCl) over a frequency range from 0.1 to 10^5^ Hz at 0.17 V. Fourier transform infrared spectra (FTIR) of the transmission type were monitored by a 380 FTIR spectrophotometer (Nicolet, Japan) at a resolution of 4 cm^−1^. In order to obtain a sufficient number of samples, the MIP and POPD samples were polymerized on the surface of indium tin oxide (ITO) electrodes, respectively. After scraping off the polymers and drying at 100 °C for 3 h, the samples were prepared by the potassium bromide pellet method. The scanning electron microscopy (SEM) was carried out using a Sigma 360 scanning electron microscope (ZEISS, Oberkochen, Germany) with an acceleration voltage of 5 kV to characterize various film electrodes. Detachable GCE electrodes were used for the preparation of SEM samples. Before SEM measurements, a thin layer of gold film was coated on the surface of the samples.

### 2.3. Pretreatment of Bare GCE

Prior to use, the surface of the bare GCE was polished by 0.3 and 0.05 mm alumina slurry. Then the GCE was washed and sonicated in ethanol and deionized water for 1 min. After drying at room temperature (RT), it was subjected to a sweep cyclically in 0.5 mM K_3_[Fe(CN)_6_] between −0.20 and +0.60 V to judge the quality of the pretreatment process.

### 2.4. Electrodeposition of AuNP-rGO Composites

After optimization based on the reported literature [25], AuNP-rGO composites were prepared as follows: GO was dispersed in distilled water and subjected to ultrasonic oscillation for 1 h to obtain a 1 mg·mL^−1^ dispersion. Then, 15 μL of the GO dispersion was transferred onto the surface of the polished GCE electrode using a pipette and dried at RT for 30 min. A three-electrode system was constructed with the GO/GCE electrode as the working electrode, which was then placed in a 1.0 mM HAuCl_4_ solution and underwent potentiostatic deposition at −1.0 V for 250 s to obtain the AuNP-rGO/GCE electrode. Finally, the obtained electrode was dried under RT and characterized by CV and EIS.

### 2.5. Preparation of MIP and Non-Molecularly Imprinted Polymer (NIP) Electrodes

After optimization, the pre-polymerization solution containing 5 mM CBD as the template and 10 mM OPD as the monomer were mixed and dispersed in 0.1 M acetate buffer solution at pH 5.2. To obtain stable and good molecularly imprinted recognition properties, the pre-polymerization solution was freshly prepared and ultrasonic agitation conducted for 1 h before being used. Then, a three-electrode system with a AuNP-rGO/GCE electrode as the working electrode was placed in the pre-polymerization solution for CV scanning from 0 to 0.8 V with a scan rate of 0.05 V·s^−1^. After 30 cycles of electropolymerization, the MIP/AuNP-rGO/GCE electrode was obtained. After drying at RT for 30 min, the MIP/AuNP-rGO/GCE electrode was placed in a 20 mL methanol/acetic acid (9:1, *V*/*V*) solution and magnetically stirred for 15 min to remove CBD. After being rinsed with distilled water and dried at RT again, the CBD-free MIP/AuNP-rGO/GCE electrode was obtained, which had a specific recognition ability for CBD. The CBD rebinding process was performed by immersing CBD-free MIP/AuNP-rGO/GCE electrodes in different CBD solutions for 15 min and then dried at RT to obtain the CBD-rebinding MIP/AuNP-rGO/GCE electrodes. The non-molecularly imprinted polymer (NIP) film electrodes were prepared in the same way mentioned above but without the addition of CBD.

## 3. Results and Discussion

### 3.1. Electropolymerization of MIP Films on AuNP-rGO/GCE Electrodes

**Figure 1:**
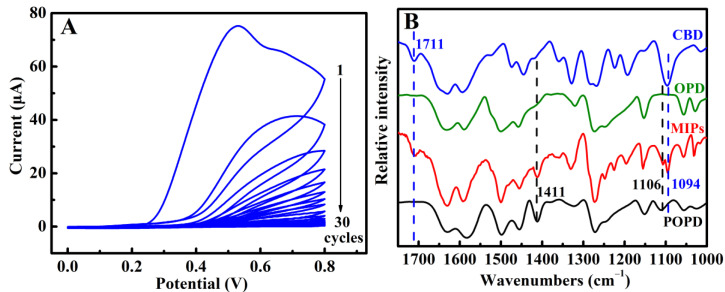
(**A**) Continuous CVs of 10 mM OPD at AuNP-rGO/GCE electrodes at 0.05 V s^−1^ in a pH 5.2 buffer solution containing 5 mM CBD. (**B**) FTIR spectra of CBD, OPD, MIP, and POPD samples.

The electropolymerization of MIP films on the surface of AuNP-rGO/GCE electrode was performed by CV scanning in the presence of CBD template and OPD monomer in acetate buffer solution (0.1 M, pH = 5.2). The highest anodic peak current corresponded to irreversible OPD oxidation was obtained in the first cycle [31], then the peak current decreased significantly during continuous cycling (Figure 1A). The tendency of decreasing current gradually decreased, and finally achieved a steady value. These results illustrated the successfully formation of an insulating MIP films on AuNP-rGO/GCE electrode that would block the access of the OPD monomer to the electrode surface. In addition, FTIR spectroscopy was utilized to further confirm the successful polymerization of the MIP films. In the FTIR spectra of the MIP films (Figure 1B, red line), the C-N-C stretching vibration peak (*ν*_C-N-C_) on the benzene ring of polymer(o-phenylenediamine) (POPD) at 1106 cm⁻¹ and the characteristic stretching vibration peak of the phenazine ring (*ν*_phenazine_) at 1411 cm⁻¹ could be observed [31,32], but they were not found in the OPD monomer (Figure 1B, green line). These illustrated that during the electropolymerization process, OPD monomer had been polymerized into POPD. Furthermore, in the infrared spectrum of MIP films, the C=O stretching vibration peak (*ν*_C=O_) of CBD at 1711 cm⁻¹ and the C-O stretching vibration peak (*ν*_C-O_) at 1094 cm⁻¹ could also be found, thus confirming that the CBD template molecules had been successfully embedded into MIP films. The measurement results mentioned above indicated that MIP films had been successfully polymerized.

### 3.2. Characterization of Film Electrodes

**Figure 2:**
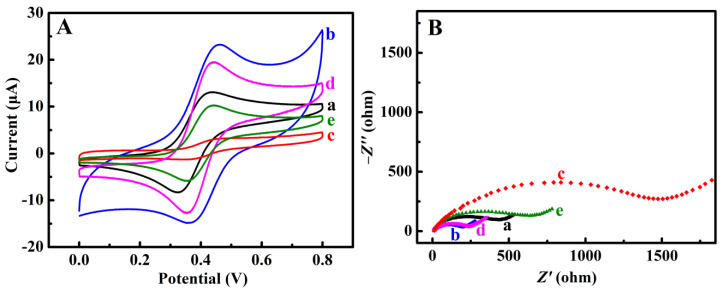
(**A**) CVs of 0.5 mM Fc(COOH)_2_ at 0.05 V s^−1^ in pH 7.0 PBS and (**B**) EIS responses of 5 mM Fe(CN)_6_^4−/3−^at 0.17 V for (a) bare GCE, (b) AuNP-rGO/GCE, (c) MIP/AuNP-rGO/GCE, (d) CBD-free MIP/AuNP-rGO/GCE, and (e) CBD-rebinding MIP/AuNP-rGO/GCE after 5 μM CBD rebinding.

Cyclic voltammetry (CV), electrochemical impedance spectroscopy (EIS), and scanning electron microscopy (SEM) measurements were deployed to characterize the construction process of the CBD MIES. The CV detection was performed with 0.5 mM Fc(COOH)_2_ as the probe, and the detection results are shown in Figure 2A. The Fc(COOH)_2_ probe exhibited reversible redox peaks on the surface of the bare GCE electrode (Figure 2A, curve a). After the electrodeposition of AuNP-rGO composites on the surface of the GCE electrode, the current response increased significantly (Figure 2A, curve b) due to the good electrical conductivity of AuNP-rGO composites and the increased electrochemical active area of GCE, which could effectively facilitate the electrochemical transfer of the Fc(COOH)_2_ probe. As depicted by curve c in Figure 2A, when a layer of MIP films was electropolymerized on the surface of the AuNP-rGO/GCE electrode, the current response of the obtained MIP/AuNP-rGO/GCE decreased remarkably. The reason was that the redox reaction of Fc(COOH)_2_ probe on GCE electrode was hindered by MIP films. After the CBD was removed, the redox peak current of Fc(COOH)_2_ increased obviously (Figure 2A, curve d), because the specific recognition sites for CBD were exposed in MIP films, and the porous MIP was conducive to the passage of Fc(COOH)_2_ probe to reach the electrode surface for electron transfer. After rebinding 5 μM CBD, the current response of the CBD-rebinding MIP/AuNP-rGO/GCE electrode decreased significantly due to the reoccupation of the specific recognition sites for CBD in the MIP films (Figure 2A, curve d). The corresponding EIS measurements for those film electrodes of the CV results described above are shown in Figure 2B.

**Figure 3:**
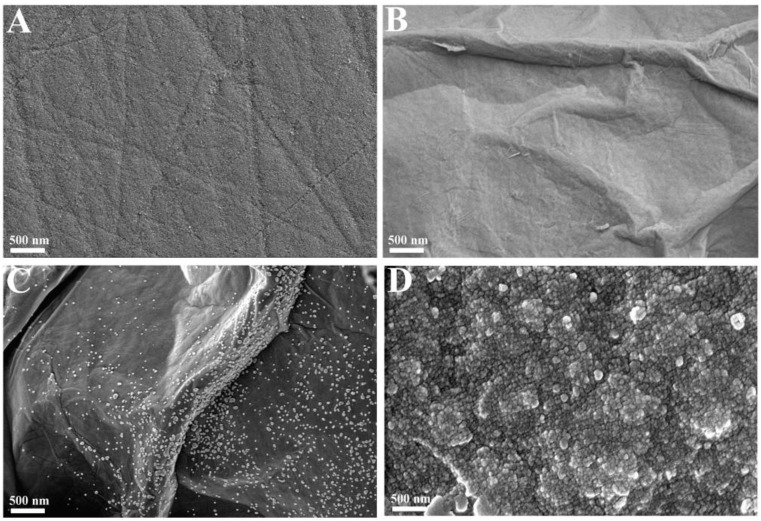
SEM top-view images of (**A**) bare GCE, (**B**) GO/GCE, (**C**) AuNP-rGO/GCE, and (**D**) MIP/AuNP-rGO/GCE.

As shown in the SEM top-view images in Figure 3, a typical crumpled and wrinkled sheet structure was distributed on the surface of the GCE after GO was coated and dried (panels A and B). After applying −1.0 V for 250 s, a layer of relatively evenly distributed nanoparticles on the surface of wrinkled sheet can be found in Figure 3, panel C, suggesting the AuNP-rGO composites were successfully prepared. The size of the nanoparticles was in the range of 20~40 nm, which could further increase the surface area of the electrode and facilitate the electron transfer rate [26]. After the electropolymerization of POPD and CBD, the morphology of the electrode surface demonstrated an obvious change with a layer of agglomerated particles, indicating the formation of AuNP-rGO/GCE (Figure 3, panel D) indicating the successful formation of MIP films on the surface of AuNP-rGO/GCE. All these results suggested the successful fabrication of MIP film electrodes.

### 3.3. Condition Optimization

#### 3.3.1. Optimization of Electropolymerization Cycles

**Figure 4:**
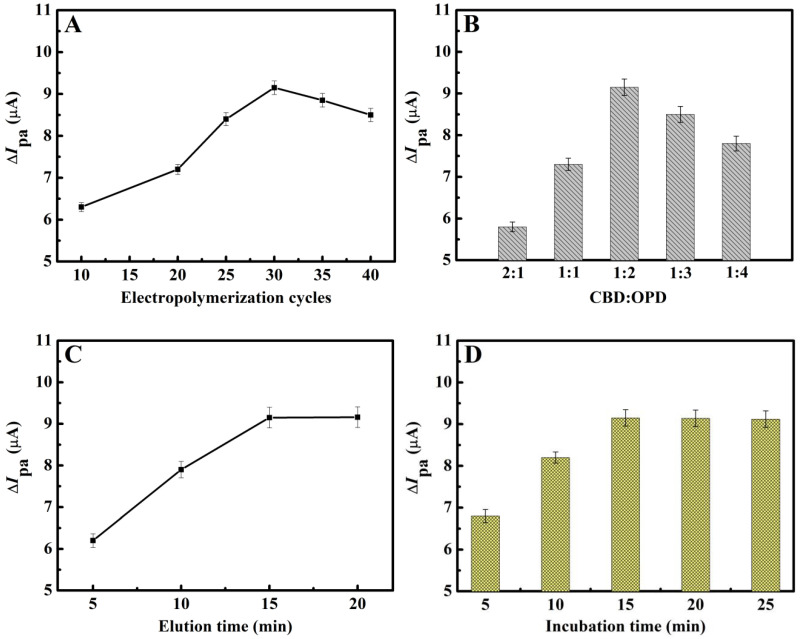
Effect of (**A**) the electropolymerization cycles, (**B**) molar ratio of CBD to OPD, (**C**) elution time, and (**D**) incubation time on the Δ*I*_pa_ of the sensor for 5 μM CBD. The CV current responses were measured in 0.5 mM Fc(COOH)_2_ at 0.05 V s^−1^ in pH 7.0 PBS. The error bars represent the standard deviation of the experimental results (*n* = 3).

The number of scan cycles during the electropolymerization process has a significant influence on the thickness of MIP films, which is very important to the sensitivity and stability of the sensor [33]. As shown in Figure 4A, when the number of cycles was increased to 30, Δ*I*_pa_ reached the maximum value. Herein, Δ*I*_pa_ was the difference between the CV oxidation peak current of 0.5 mM Fc(COOH)_2_ at a scan rate of 0.05 V s^−1^ for CBD-free and CBD-rebinding MIP/AuNP-rGO/GCE electrodes with 5 μM CBD. With the further increasing of cycle number, Δ*I_pa_* decreased. The reasons were as follows: the MIP films were relatively thin and unstable when the scan cycles numbered less than 30. And the number of specific recognition sites in the MIP films and the thickness of the MIP films increased with the increasing of the numbers of polymerization cycles. However, overly thick MIP films would exacerbate the difficulty of template removal process, and reduce the number of effective specific recognition sites in MIP films. Therefore, 30 cycles were the optimum scan cycles during the electropolymerization process.

#### 3.3.2. Optimization of the Ratio of CBD to OPD

The ratio of template molecules to functional monomers also has a significant impact on the number of specific recognition sites in the MIP films, thereby exerting a profound influence on the recognition ability of the MIP films [34]. In the present work, CBD-free MIP/AuNP-rGO/GCE electrodes with different molar ratios of CBD to OPD were applied to detect 5 μM CBD. As shown in Figure 3B, when the ratio was 1:2, Δ*I*_pa_ reached its maximum value. When the ratio was 2:1 and 1:1, the amount of functional monomer was too small to bind enough the template molecules, thus reducing the number of available binding sites and decreasing the electrochemical responsive of recognition. When the ratio was 1:3 and 1:4, Δ*I*_pa_ was also lower than the optimum current response, the reason might be that the number of functional monomers was too large, occupying the electrode surface area and restricting the quantity of available binding sites. Thus, the molar ratio of 1:2 was selected as the optimal ratio of CBD to OPD.

#### 3.3.3. Effect of Time for CBD Removal and Rebinding

In order to find the best time for CBD removal and improve the recognition ability of the sensor, the MIP/AuNP-rGO/GCE electrodes were placed in a 20 mL methanol/acetic acid (9:1, *V/V*) solution and magnetically stirred for 5, 10, 15, and 20 min to remove CBD. As shown in Figure 3C, the maximum current response for 5 μM CBD detection was obtained when the time for CBD removal was 15 min, and the current response reached a plateau from 15 to 20 min. Therefore, the best time for CBD removal was 15 min in this work.

To examine the influence of incubation time on the current response of the sensor, the imprinted sensor was incubated in 5 μM CBD solution for different time at RT. As shown in Figure 3D, the current response increased with the increasing incubation time and reached a plateau at 15 min. Therefore, the incubation time of 15 min was chosen.

### 3.4. Amplification by Bioelectrocatalysis in Determination of CBD

**Figure 5:**
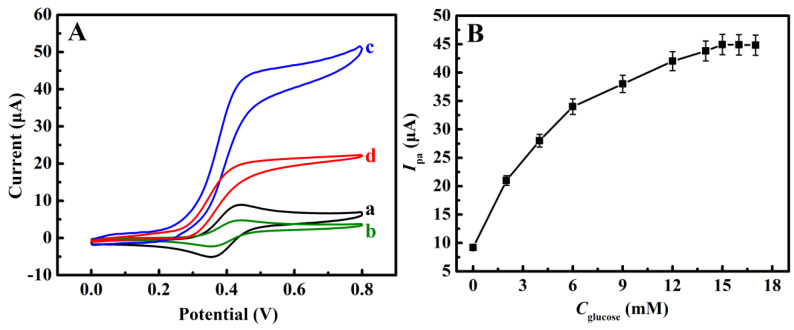
(**A**) CVs of 0.5 mM Fc(COOH)_2_ at 0.01 V s^−1^ in pH 7.0 PBS at (a) CBD-free MIP/AuNP-rGO/GCE, (b) CBD-rebinding MIP/AuNP-rGO/GCE after 5 μM CBD rebinding, (c) CBD-free MIP/AuNP-rGO/GCE in the presence of 1 mg mL^−1^ GOD and 15 mM glucose, and (d) CBD-rebinding MIP/AuNP-rGO/GCE after 5 μM CBD rebinding in the presence of 1 mg mL^−1^ GOD and 15 mM glucose. (**B**) CV *I*_pa_ of 0.5 mM Fc(COOH)_2_ at 0.01 V s^−1^ in pH 7.0 PBS containing 1 mg mL^−1^ GOD and different concentrations of glucose (*C*_glucose_). The CV measurements were performed under a nitrogen atmosphere and the error bars represent the standard deviation of the experimental results (*n* = 3).

The electrochemical redox process of glucose, catalyzed by GOD and mediated by Fc(COOH)_2_, was utilized to amplify the detection of antibiotics in the literature [11]. In this work, this amplification strategy was applied to improve the oxidation peak current of the Fc(COOH)_2_ probe to detect CBD. As shown in Figure 5A, the CBD-free MIP/AuNP-rGO/GCE electrode was placed in the Fc(COOH)_2_ probe solution for CV measurement, and a pair of nearly reversible redox peaks was obtained (curve a). After rebinding the CBD, both the oxidation and reduction currents of this pair of redox peaks were decreased (Figure 5A, curve b). The obtained CV Δ*I*_pa_ for 5 μM CBD determination was only 4.09 μA.

When 1 mg mL^−1^ glucose oxidase (GOD) and 15 mM glucose (the optimization experiments are shown in Figure 5B) were added to the Fc(COOH)_2_ probe solution after being bubbled with high-purity nitrogen for at least 10 min, the oxidation peak current of Fc(COOH)_2_ increased significantly accompanied by a gradual decrease in the reduction peak current (Figure 5A, curve c). After rebinding of 5 μM CBD, the oxidation peak of Fc(COOH)_2_ on the CBD-rebinding MIP/AuNP-rGO/GCE electrode also decreased (Figure 5A, curve d). The obtained CV Δ*I*_pa_ for CBD determination was 25.4 μA, indicating that under the amplification effect of bioelectrocatalysis, Δ*I*_pa_ was amplified by 6.2 times compared those without GOD and glucose (Figure 5A, curves a and b). Thus, CV technique, after optimization, effectively recorded this detection under amplification of the bioelectrochemical catalysis. The amplification mechanism of this bioelectrocatalytic system was shown in the following equations [35,36]:

GOD(FAD) + glucose → GOD(FADH_2_) + gluconolactone (1)

GOD(FADH_2_) + 2Fc(COOH)_2Ox_ → GOD(FAD) + 2Fc(COOH)_2Red_ (2)

Fc(COOH)_2Red_ − e^−^ ⇔ Fc(COOH)_2Ox_   at electrode (3)

Here, GOD(FAD) and GOD(FADH_2_) represent the oxidized and reduced forms of GOD, respectively. The amplification function of the bioelectrocatalytic system could be applied to improve the detection sensitivity of the CBD imprinted electrochemical sensor.

### 3.5. Calibration Curve

**Figure 6:**
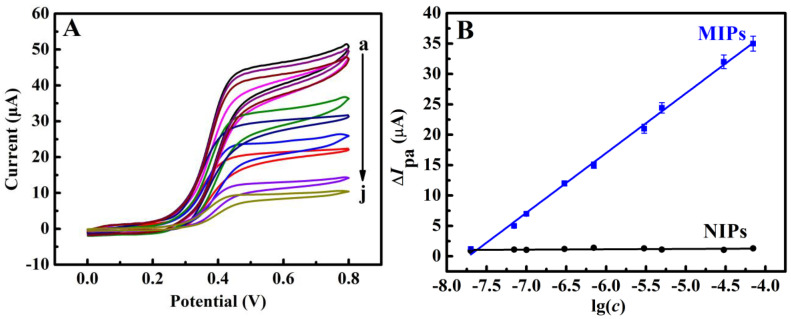
(**A**) CVs of 0.5 mM Fc(COOH)_2_ at 0.01 V s^−1^ in pH 7.0 PBS containing 1 mg mL^−1^ GOD and 15 mM glucose for CBD-rebinding MIP/AuNP-rGO/GCE after rebinding CBD at different concentrations (a)–(j): 0, 0.02, 0.07, 0.1, 0.3, 0.7, 3, 5, 30, and 70 μM. (**B**) Calibration curves for the MIP and NIP film electrodes. The CV measurements were performed under a nitrogen atmosphere and the error bars represent the standard deviation of the experimental results (*n* = 3).

The analytical performance of the CBD-imprinted electrochemical sensor for CBD detection was investigated. The CBD-free MIP/AuNP-rGO/GCE electrodes were placed in CBD solutions with a CBD concentration from 5.0 × 10^−11^ to 5.0 × 10^−3^ M for 15 min to obtain CBD-rebinding MIP/AuNP-rGO/GCE electrodes. Then, different concentrations of CBD-rebinding MIP/AuNP-rGO/GCE electrodes were placed in pH 7.0 PBS containing 0.5 mM Fc(COOH)_2_, 1 mg mL^−1^ GOD, and 15 mM glucose to perform CV measurements at 0.01 V s^−1^. As shown in Figure 6A, the CV oxidation peaks continuously decreased with the increase of the concentration of rebinding CBD. The quantitative determination of CBD could be performed from the relationship between Δ*I*_pa_ and the CBD concentration (*C*), where Δ*I*_pa_ is the difference between the CV oxidation peak current of the Fc(COOH)_2_ probe at CBD-free and CBD-rebinding MIP/AuNP-rGO/GCE electrodes. The standard curve of Δ*I*_pa_ to lg(*c*) can be described by the following equation: Δ*I*_pa_ (μA) = 75.9 (μA) + 9.82 lg(*c*) (r = 0.997); the linear range was from 2.0 × 10^−9^ to 7.0 × 10^−5^ M as shown in Figure 5B. The limit of detection (LOD) was 0.68 nM (S/N = 3). The comparison of the MIESs constructed in the present work with other electrochemical sensors in the literature is shown in Table 1 [18,37,38,39,40,41,42]. For the NIP film electrodes, the CV *I*_pa_ values were found to be small and did not exhibit any significant changes after the detection of CBD with different concentrations (Figure 6B). This was due to the fact that the NIP films had no specific recognition sites for CBD and could not recognize CBD.

**Figure 7:**
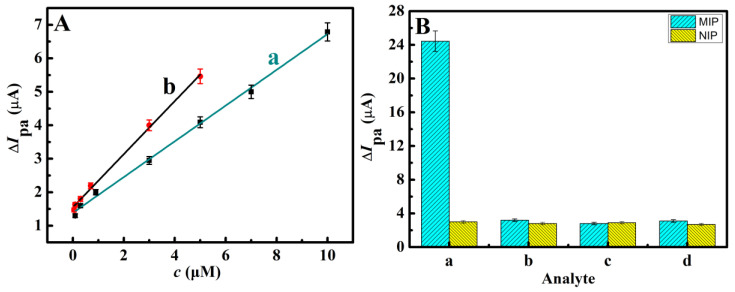
(**A**) Calibration curves for (a) sensor A that only modified AuNP-rGO composites and (b) sensor B that only utilized the bioelectrocatalysis. (**B**) CV Δ*I_pa_* of selective recognition for the CBD-imprinted and NIP sensors. (a) 5 μM CBD, (b) 5 mM TBZ, (c) 5 mM TPM, and (d) 5 mM 2-ABZ. The CV measurements were performed under a nitrogen atmosphere and the error bars represent the standard deviation of the experimental results (*n* = 3).

In order to study the effect of the synergistic amplification of bioelectrocatalysis and nanocomposites, the analytical properties of sensors that only modified AuNP-rGO composites (named as sensor A) and only utilized the bioelectrocatalysis (named as sensor B) were investigated for comparison. The preparation processes of sensor A were the same as the sensor constructed in this paper, except that neither GOD nor glucose existed in the Fc(COOH)_2_ probe solution. As shown in Figure 7A, curve a, the linear range of sensor A was from 9.0 × 10^−8^ to 1.0 × 10^−5^ M. The fitting curve of Δ*I*_pa_ on *c* was shown as follows: Δ*I*_pa_ (μA) = 1.36 (μA) + 0.54 *c* (*r* = 0.994), with the LOD of 7.7 × 10^−8^ M (S/N = 3). For sensor B, the MIP films were directly prepared by an electropolymerization method on the surface of bare GCE electrode without nanocomposites and the detection process was carried out in an Fc(COOH)_2_ probe solution containing both GOD and glucose. The linear range was from 5.0 × 10^−8^ to 5.0 × 10^−6^ M (Figure 7A, curve b) with a linear equation of sensor B of Δ*I*_pa_ (μA) = 1.55 (μA) + 0.79 *c* (*r* = 0.997) and LOD of 2.9 × 10^−8^ M (S/N = 3). Therefore, with the synergistic effect of bioelectrocatalysis and nanocomposites, the proposed sensor in this work exhibited a near 2 magnitude of lower LOD and higher sensitivity compared with sensors A and B (Figure 6B and Figure 7A).

### 3.6. Selectivity of the Sensor

Three CBD analogues including thiabendazole (TBZ), thiophanate methyl (TPM), and 2-aminobenzimidazole (2-ABZ) were selected to investigate the selectivity performance of the sensor. As shown in Figure 7B, the sensor showed a considerably greater response to 5 μM CBD than to those three analogues (5 mM) because specific recognition sites that could recognize complementary to CBD in terms of the size and shape were formed in MIP films after the removal of CBD, and the structure of other three analogues cannot match with the recognition sites. As a control experiment, the NIP films exhibited a very low signal response to both CBD and the other three analogues. The detection results of the selectivity experiment demonstrated that the sensor exhibited excellent selectivity to CBD.

### 3.7. Repeatability, Reproducibility, Precision, and Stability of the Sensor

Repeatability: the repeatability of the sensor was tested by using a CBD-imprinted sensor to detect the current response of 5 μM CBD solution repeatedly. The relative standard deviation (RSD) was 4% (*n* = 3). It indicated that the CBD-imprinted sensor had a good repeatability.

Reproducibility and precision: three CBD-imprinted sensors were prepared under the same conditions to research the reproducibility of the sensor by detecting 5 μM CBD solution. The precision of the described procedure in terms of RSD was 5%. Thus, the sensor had good reproducibility and precision in the detection of CBD.

Stability: multiple groups of CBD-imprinted sensors were prepared and stored at RT, each group consisting of three parallel samples (*n* = 3). One group was performed once a day to detect 5 μM CBD. It was found that after being stored for 10 d, the current response decreased by approximately 7% compared with the first day, which suggested that the developed CBD-imprinted sensor possessed good stability.

### 3.8. Determination of CBD in Real Samples

In order to ascertain the potential application of the CBD-imprinted sensor, the content of CBD in real samples was analyzed by the sensor. Grape juice and apple juice samples were purchased from the local market. They were diluted by deionized water and filtered through a sterile millipore membrane (0.22 μm). To verify the accuracy of the sensor in real sample detection, different concentrations of CBD (5.00 × 10^−6^, 5.00 × 10^−7^, and 5.00 × 10^−8^ M) were added into the samples for determination. The sample detection results in spiked grape juice and apple juice samples involving CBD are shown in Table 2. Each sample had three parallel measurements. The recoveries were from 97.8% to 103.6%, which indicated that the sensor could be successfully used to detect CBD in real samples.

## 4. Conclusions

In this work, a highly sensitive and selective MIES for CBD detection was developed. The AuNP-rGO composites were prepared by a one-step electrodeposited method and enhanced the electrochemical response of the sensor due to the accelerated electron transfer and excellent conductivity by AuNP and rGO. The MIP films were prepared on AuNP-rGO/GCE electrodes by an electropolymerization method with CBD as the template molecule and OPD as the functional monomer. Through optimization of electropolymerization cycles, CBD to OPD molar ratio, and the time of CBD removal and incubation, the sensor achieved excellent performance. The bioelectrocatalysis of the Fc(COOH)_2_-GOD-glucose system amplified the detection signal of Fc(COOH)_2_ probe, thus further improving the detection sensitivity of the sensor. The sensor exhibited a wide linear range, relatively low LOD, high selectivity, good repeatability, reproducibility, precision, and stability. Successful detection of CBD in real samples demonstrated its practical applicability. The strategy in this work is universal and general, in which different nanocomposites and bioelectrocatalytic systems with different enzymes can be used to improve the detection sensitivity of the sensor. This work might offer a novel approach for developing sensitive MIES, with potential for further applications in pesticide residue analysis.

## Figures and Tables

**Table 1:** Comparison of the analytical performance for CBD detection in this work with other electrochemical sensors.

Sensor Type ^a^	Electrode ^b^	Method ^c^	Linear Range (M)	LOD (M)	Ref.
MIES	N,S-Mo_2_C/GCE	CV	1.0 × 10^−12^~8.0 × 10^−9^	6.7 × 10^−13^	[18]
ES	TT-COF/GCE	DPV	5.0 × 10^−9^~5.0 × 10^−6^	2.21 × 10^−9^	[37]
ES	Co@Mo_2_C/GCE	SWV	1.0 × 10^−8^~1.0 × 10^−3^	3.4 × 10^−9^	[38]
MIES	HKUST-1/GE	DPV	1.0 × 10^−8^~5.0 × 10^−5^	2.0 × 10^−9^	[39]
MIES	GQDs/GECE	DPV	2.0 × 10^−7^~4.5 × 10^−5^	1.0 × 10^−9^	[40]
ES	[Cu(adp)(BIB)(H_2_O)]n/GCE	DPV	1.0 × 10^−7^~1.0 × 10^−5^	5.0 × 10^−8^	[41]
ES	Au@Pt/CNHs@RGO/GCE	DPV	5.0 × 10^−8^~5.0 × 10^−5^	1.64 × 10^−9^	[42]
MIES	AuNP-rGO/GCE	CV	9.0 × 10^−8^~1.0 × 10^−5^	7.70 × 10^−8^	Sensor A in this work
MIES	GCE	CV	5.0 × 10^−8^~5.0 × 10^−6^	2.90 × 10^−8^	Sensor B in this work
MIES	AuNP-rGO/GCE	CV	2.0 × 10^−9^~7.0 × 10^−5^	6.80 × 10^−10^	Proposed sensor in this work

^a^ ES: electrochemical sensor. ^b^ GE: graphite-epoxy electrode; GECE: graphite-epoxy composite electrode. ^c^ DPV: differential pulse voltammetry; SWV: square wave voltammetry.

**Table 2:** Determination of CBD in real spiked samples (*n* = 3).

Samples	Found (M)	Added (M)	Total Found (M)	Recovery (%)
Grape juice	Not detected	5.00 × 10^−6^	5.18 × 10^−6^	103.6
	Not detected	5.00 × 10^−7^	4.92 × 10^−7^	98.4
	Not detected	5.00 × 10^−8^	5.17 × 10^−8^	103.4
Apple juice	Not detected	5.00 × 10^−6^	4.89 × 10^−6^	97.8
	Not detected	5.00 × 10^−7^	5.14 × 10^−7^	102.8
	Not detected	5.00 × 10^−8^	4.96 × 10^−8^	99.2

## Data Availability

Data are contained within the article.
